# A dimerized urea-based inhibitor of the prostate-specific membrane antigen for ^68^Ga-PET imaging of prostate cancer

**DOI:** 10.1186/2191-219X-2-23

**Published:** 2012-06-06

**Authors:** Martin Schäfer, Ulrike Bauder-Wüst, Karin Leotta, Frederic Zoller, Walter Mier, Uwe Haberkorn, Michael Eisenhut, Matthias Eder

**Affiliations:** 1 Radiopharmaceutical Chemistry, German Cancer Research Center, Im Neuenheimer Feld 280, Heidelberg, 69120, Germany; 2 Department of Nuclear Medicine, University of Heidelberg, Im Neuenheimer Feld 400, Heidelberg, 69120, Germany

**Keywords:** Dimerization, ^68^Ga-PET imaging, PSMA, HBED-CC, Prostate cancer

## Abstract

**Background:**

Alternative positron-emission tomography (PET) probes like labeled inhibitors of the prostate-specific membrane antigen (PSMA) are of emerging clinical impact as they show the ability to image small lesions of recurrent prostate cancer. Here, the dimerization of the pharmacophore Glu‐ureido‐Lys via the ^68^Ga chelator *N,N′*-bis[2-hydroxy-5-(carboxyethyl)benzyl]ethylenediamine-*N,N′*-diacetic acid (HBED-CC) was investigated to further improve the binding characteristics and pharmacokinetics.

**Methods:**

The peptidomimetic structures were synthesized by solid-phase chemistry, and the resulting products were coupled with the respective 2,3,5,6-tetrafluorophenol esters of HBED-CC to form the monomeric reference and the dimeric Glu‐ureido‐Lys derivative. The binding properties were analyzed in competitive binding, internalization, and cell surface retention experiments. PET images and biodistribution data were obtained 1 h after injection in BALB/c nu/nu mice bearing LNCaP tumor xenografts.

**Results:**

Cell binding data revealed significant better binding properties of the dimer (IC_50_ = 3.9 ± 1.8 nM; IC_50_ (monomer) = 12.1 ± 2.1 nM). The inhibition potency investigated by the enzyme-based NAALADase assay confirmed these results. Specific internalization in LNCaP cells was demonstrated for both, the monomer and dimer. As shown by efflux measurements, the dimeric compound was more effectively retained on the cell surface, resulting in advanced *in vivo* properties (T/B_Monomer_ = 9.2; T/B_Dimer_ = 26.5).

**Conclusions:**

The dimeric [^68^Ga]7 is a promising imaging agent for PSMA-expressing tumors as it shows higher tumor uptake while observing more favorable background clearance. As compared to the respective monomer, the higher affinity and prolonged tumor retention additionally represent promising features and warrant further evaluation regarding ^68^Ga-PET imaging of PSMA expression.

## Background

The occurrence of metastases is one of the major causes of morbidity and mortality in prostate cancer patients. The early detection of these metastatic or recurrent lesions is of high clinical relevance for staging, prognosis, and therapy management [1,2]. In order to improve prostate cancer imaging, a series of ^11^ C- and ^18^ F-labeled choline and acetate derivatives have been developed [3-7]. However, with regard to the detection of small lesions of recurrent prostate cancer, these radiopharmaceuticals were shown to be limited in detecting lymph node metastases after radical retropubic prostatectomy in patients with low PSA level [8]. For example, ^18^ F]fluoroethylcholine positron-emission tomography (PET)/CT only showed 71% sensitivity in the detection of recurrent prostate cancer [9] which gave rise to the development of alternative PET probes with more specific targeting characteristics.

The prostate-specific membrane antigen (PSMA) represents a cell surface target suitable for imaging metastatic lesions as it is expressed by nearly all prostate cancer cells with enhanced expression levels in poorly differentiated, metastatic, and hormone-refractory carcinomas [10,11]. Low levels of physiologic PSMA expression were detected in the brain, kidney, spleen, liver, and small intestine [10], whereas other human tissues showed no PSMA expression at all [12-14]. PSMA-specific tracers can be considered as ideal for imaging small lesions of recurrent or metastasized prostate cancer since the PSMA expression even increases with cancer progression [11]. Furthermore, they are capable to visualize prostate cancer cells independent of proliferation status [15-17].

Urea-based inhibitors of PSMA clear rapidly from the circulation, leading to images with clear contrast [18-23]. Further improvement of PSMA inhibitors regarding affinity, specificity, and radiolabeling may lead to significant clinical benefits as lesions could be detected earlier and convenient kit-like radiolabeling makes a tracer more accessible.

It has been previously reported that dimeric peptides such as cyclic arginine-glycine-aspartic acid (RGD) exhibit significant higher tumor uptake and prolonged retention as compared to their corresponding monomeric analogues [24-28]. Especially, the enhancement of tumor retention results in higher contrast and opens the possibility of using small molecules for therapy. In this context, we synthesized a PSMA-selective dimer labeled with the ^68^Ga complex of *N,N′*-bis[2-hydroxy-5-(carboxyethyl)benzyl]ethylenediamine-*N,N′*-diacetic acid (HBED-CC) which was recently proposed for efficient radiolabeling with ^68^Ga at ambient temperature [29,30]. A convenient labeling procedure combined with the usage of ^68^Ga might additionally improve prostate cancer imaging. Furthermore, the lipophilic ^68^Ga complex of HBED-CC proved to be favorable for the linker region, a feature which was found to be necessary for a sustainable interaction with the PSMA ‘active binding site’ [21,31,32]. As a consequence, the HBED-CC-labeled monomeric reference compound showed promising preclinical results with high potential for clinical translation [32,33]. Here, we present the synthesis of the dimer, cell-based *in vitro* binding data, and preliminary *in vivo* results.

## Methods

All commercially available chemicals were of analytical grade and were used without further purification. ^68^Ga (half-life 68 min, *β*^+^ 89%, *E*_β+_ max. 1.9 MeV) was obtained from a ^68^Ge/^68^Ga generator based on pyrogallol resin support [30,34]. Typically, 1 GBq ^68^Ga was eluted using 5.5 M HCl. The activity was trapped on a small anion-exchanger cartridge (AG1X8, Biorad, Richmond, CA, USA) as ^68^Ga]GaCl_4_^−^. The radiogallium was eluted from the cartridge in a final volume of 300 μL of ultrapure water (Merck, Darmstadt, Germany) as ^68^Ga]GaCl_3_. ^67^Ga was purchased from MDS Nordion (Fleurus, Belgium) as ^67^Ga]GaCl_3_ in 0.1 N HCl. Protected amino acids were purchased from Novabiochem (Merck, Darmstadt, Germany) or Sigma-Aldrich (Neu-Ulm, Germany).

The compounds were analyzed by reversed-phase high-performance liquid chromatography (RP-HPLC; Chromolith RP-18e, 100 × 4.6 mm; Merck, Darmstadt, Germany). Analytical HPLC runs were performed using a linear A-B gradient (0% B to 100% B in 6 min) at a flow rate of 4 mL/min. Solvent A consisted of 0.1% aqueous trifluoroacetic acid (TFA), and solvent B was 0.1% TFA in CH_3_CN or MeOH (in case of determination of radiochemical yield (RCY) or analysis of serum stability).

The HPLC system (L6200 A; Merck-Hitachi, Darmstadt, Germany) was equipped with a variable UV and a gamma detector (Bioscan, Washington, DC, USA). UV absorbance was measured at 214 and 254 nm. Mass spectrometry was performed with a MALDI-MS Daltonics Microflex (Bruker Daltonics, Bremen, Germany) system. ESI-MS analysis was performed using an Orbitrap Mass Spectrometer (Exactive, Thermo Fisher Scientific, Dreieich, Germany). Full-scan single mass spectra were obtained by scanning from *m*/*z* = 200 to 4,000.

### Synthesis of protected Glu‐ureido‐Lys(Ahx) (5)

**Figure 1:**
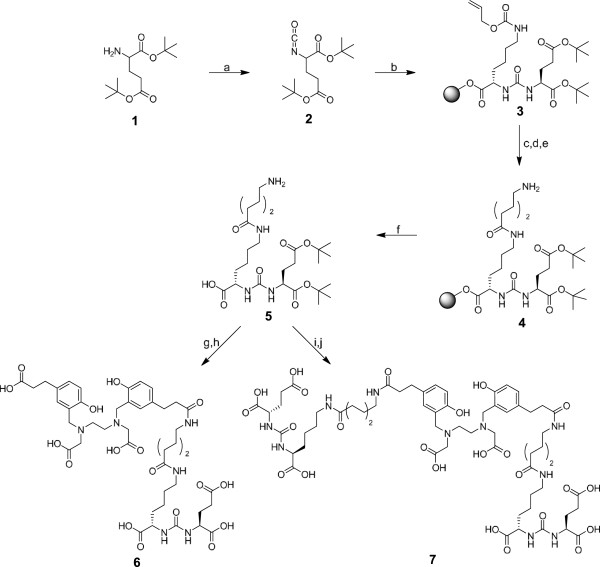
**Syntheses of Glu‐ureido‐Lys(Ahx)-HBED-CC (6) and [Glu‐ureido‐Lys(Ahx)]**_**2**_**-HBED-CC (7).** (**a**) Triphosgene, DIPEA, CH_2_Cl_2_, 0°C; (**b**) H-Lys(Alloc)-2CT-Resin, CH_2_Cl_2_; (**c**) Pd[P(C_6_H_5_)_3_]_4_, morpholine, CH_2_Cl_2_; (**d**) Fmoc-6-Ahx-OH, HBTU, DIPEA, DMF; (**e**) 20% piperidine, DMF; (**f**) hexafluoroisopropanol/CH_2_Cl_2_; (**g**) HBED-CC-TFP ester, DIPEA, DMF; (**h**) TFA; (**i**) (HBED-CC)TFP_2_ diester, DIPEA, DMF; (**j**) TFA.

The binding motif was synthesized as previously described [32]. Briefly, the synthesis started with the formation of the isocyanate 2 (Figure 1) of the glutamyl moiety by using triphosgene. A resin-immobilized (*2*-chloro-tritylresin, Merck, Darmstadt, Germany), ε-allyloxycarbonyl-protected lysine was added and reacted for 16 h with gentle agitation resulting in compound 3. The resin was filtered off, and the allyloxycarbonyl-protecting group was cleaved. The coupling of the aminohexanoic linker was performed using fluorenylmethyloxycarbonyl (Fmoc)-protected 6-aminohexanoic acid (Sigma-Aldrich, Germany). The product 4 was cleaved from the resin with 30% *1,1,1,3,3,3*-hexafluoroisopropanole in CH_2_Cl_2_, resulting in the tert-butyl-protected product 5. Purification was done using a Chromolith RP-18e column (100 × 10 mm; Merck, Darmstadt, Germany) with a gradient in 6 min starting at 0% B, raised to 60% B and followed by a 1-min increase to 100% B. Solvent A consisted of 0.1% aqueous TFA, and solvent B was 0.1% TFA in CH_3_CN. The flow rate was 6 mL/min.

The diastereomeric d-isomer (d-Glu‐ureido‐Lys(Ahx)) was synthesized using protected d-glutamate instead of l-glutamate in the first step. The further synthesis and purification was performed as described for the l-isomer.

### Synthesis of the HBED-CC-conjugated monomer (6)

To conjugate HBED-CC, the purified product 5 was reacted with 1.2 Eq of HBED-CC-2,3,5,6-tetrafluorophenol (TFP) ester (synthesized as previously described [30]) in the presence of 2 Eq of *NN*-diisopropylethylamine (DIPEA) in *NN*-dimethylformamide (DMF). After HPLC purification (*vide supra*), the tert-butyl groups were removed by TFA treatment at room temperature for 1 h, resulting in the monomeric structure 6 (approximately 37% yield). Mass spectrometry was used to confirm the identity of the compound (Table 1).

**Table 1:** Analytical and PSMA-binding data

**Ga-peptide complexes**	***m/z***^**a**^	***m/z*calculated as [M + H]**^**+**^	**AnalyticalHPLC retention (min)**^**b**^	**Affinity-related IC**_**50**_**determined in enzyme-based assay (nM)**^**c**^	**Affinity-related IC**_**50**_**determined in cell-based assay (nM)**^**c**^
[Ga]**6**	947.4257	947.4250	3.1	9.0 ± 1.1	12.1 ± 2.1
[Ga]**7**	1,361.6387	1,361.6364	3.1	2.1 ± 1.4	3.8 ± 1.8

^a^High-resolution mass spectrometry data of the free ligands ([M + H]^+^); ^b^compounds were labeled with ^68^Ga; runs were performed using a linear A-B gradient (0% B to 100% B in 6 min) at a flow rate of 4 mL/min; solvent A was 0.1% aqueous TFA, and solvent B was MeOH; ^c^compounds were complexed with ^nat^Ga.

### Syntheses of (HBED-CC)TFP_2_ and the HBED-CC-conjugated dimer (7)

Active ester formations were performed in accordance to previous protocols for the preparation of the monoactive ester [30]. To synthesize the bis-activated ester (HBED-CC)TFP_2_, 1,000 μL of a 0.01 M [Fe(HBED-CC)]^−^ solution in DMF was supplemented with 10 Eq of TFP and 4 Eq of *N,N*'-diisopropylcarbodiimide. After 3 days at room temperature, the product [Fe(HBED-CC)]TFP_2_ was purified by preparative HPLC. After dilution with water, the iron complex of the bis-activated ester was trapped on a preconditioned RP_18_ cartridge (Waters SepPak-Classic C18; Waters Corporation, Milford, MA, USA). To remove complexed Fe^3+^, the cartridge was flushed with 3 mL of 1 M HCl and washed with 4 mL H_2_O. The remaining (HBED-CC)TFP_2_ ester was eluted with 2 mL CH_3_CN and evaporated to dryness. Based on a HPLC standard (known concentration of (HBED-CC)TFP_2_, the isolated yield amounted to 40% to 50%. MALDI-MS: *m*/*z* = 830.0 (calculated for [M + H]^+^ C_32_H_33_F_4_N_2_O_10_ 829.7).

To form the dimer of Glu‐ureido‐Lys(Ahx), the bis-activated (HBED-CC)TFP_2_ was reacted with 2.4 Eq of the purified product 5 in the presence of 2.4 Eq of DIPEA in DMF. After HPLC purification (*vide supra*), the tert-butyl groups were cleaved at room temperature for 1 h using TFA. Compound 7 was received with approximately 32% yield after HPLC purification. Mass spectrometry confirmed the identity (Table 1).

### ^68^Ga labeling

Glu‐ureido‐Lys(Ahx)-HBED-CC (6, 0.1 to 1 nmol in 0.1 M 4-(2-hydroxyethyl)-1-piperazineethanesulfonic acid (HEPES) buffer, pH = 7.5, 100 μL) or [Glu‐ureido‐Lys(Ahx)]_2_-HBED-CC (7, 0.1 to 1 nmol in 0.1 M HEPES buffer, pH = 7.5, 100 μL) was added to a mixture of 10 μL of HEPES solution (2.1 M) and 20 μL of [^68^Ga]Ga^3+^ eluate (50 to 100 MBq). The pH of the labeling solution was adjusted to 4.2 using 30% NaOH. The reaction mixture was incubated at room temperature for 2 min. The RCY was determined via analytical RP-HPLC.

### ^67^Ga labeling

Typically, 0.1 nmol of 6 or 7 (in 0.1 M HEPES buffer, pH = 7.5, 100 μL) was added to 10 μL of HEPES solution (2.1 M), 2 μL of 1 N HCl, and 10 μL of [^67^Ga]GaCl_3_ (approximately 100 MBq) in 0.1 N HCl, resulting in a solution with a pH of 4.2. The reaction mixture was incubated for 2 min at ambient temperature. The RCY was determined via RP-HPLC.

### ^nat^Ga complexes

A 10-fold molar excess of Ga(III)-nitrate (Sigma-Aldrich) in 0.1 N HCl (10 μL) was reacted with compounds 6 or 7 (1 mM in 0.1 M HEPES buffer, pH = 7.5, 40 μL) in a mixture of 10 μL of HEPES solution (2.1 M) and 2 μL of 1 N HCl for 2 min at 80°C. The pH of the labeling solution was 4.2.

### Radiochemical stability

The radiochemical stability was analyzed in both phosphate-buffered saline (PBS) and human serum. After 2 h of incubation at 37°C, an equal volume of CH_3_CN was added to the samples to precipitate serum proteins. Subsequently, the samples were centrifuged for 5 min at 13,000 rpm. Aliquots of the supernatant and the PBS sample were analyzed via RP-HPLC. In addition, serum samples were run on a Superdex 75 GL 5/150 gel filtration column (GE Healthcare, Munich, Germany) to analyze potential protein binding (PBS pH 7 as eluent).

To analyze the complex stability against human transferrin, a 400-μL aliquot was added to 250 μg of *apo*-transferrin in PBS at pH 7 and incubated at 37°C (water bath) for 2 h. The complex stability was determined using a Superdex 75 GL 5/150 short column with PBS pH 7 as eluent.

### NAALADase assay

Recombinant human PSMA (rhPSMA, R&D Systems, Wiesbaden, Germany) was diluted in an assay buffer (50 mM HEPES, 0.1 M NaCl, pH = 7.5) to 0.4 μg/mL. The substrate Ac-Asp-Glu (Sigma, Taufkirchen, Germany; 40 μM final concentration) was mixed with [^nat^Ga]6 or [^nat^Ga]7 at concentrations ranging from 0.05 to 1,000 nM in a final volume of 125 μL of assay buffer. The mixtures were combined with 125 μL of the rhPSMA solution (0.4 μg/mL) and incubated for 1 h at 37°C. The reaction was stopped by heating at 95°C for 5 min. A 15-mM solution of ortho-phthaldialdehyde (250 μL) was added to all vials and incubated for 10 min at ambient temperature. Finally, 200 μL of the reaction solutions was loaded onto an F16 Black Maxisorp Plate (Nunc, Langenselbold, Germany) and read at excitation and emission wavelengths of 330 and 450 nm, respectively, using a microplate reader (DTX-880, Beckman Coulter, Krefeld, Germany). The data were analyzed using a one-site total binding regression algorithm of GraphPadPrism (GraphPad Software, San Diego, CA, USA).

### Cell culture

For binding studies and *in vivo* experiments, PSMA^+^ LNCaP cells (metastatic lesion of human prostatic adenocarcinoma, ATCC CRL-1740) and PSMA^−^ PC-3 cells (bone metastasis of a grade IV prostatic adenocarcinoma, ATCC CRL-1435) were cultured in DMEM medium supplemented with 10% fetal calf serum and 2 mmol/L l-glutamine (all from Invitrogen, Carlsbad, CA, USA). During cell culture, cells were grown at 37°C in an incubator with humidified air, equilibrated with 5% CO_2_. The cells were harvested using trypsin-ethylenediaminetetraacetic acid (trypsin-EDTA; 0.25% trypsin, 0.02% EDTA, all from Invitrogen) and washed with PBS.

### Cell binding and internalization

In order to determine the binding affinity, a competitive cell binding assay was performed. LNCaP cells (10^5^/well) were incubated with a 0.2-nM solution of [^67^Ga]6 in the presence of 12 different concentrations of [^nat^Ga]6 or [^nat^Ga]7 (0 to 5,000 nM, 200 μL/well). After incubation at ambient temperature for 1 h with gentle agitation, the binding buffer was removed using a multiscreen vacuum manifold (Millipore, Billerica, MA, USA). After washing twice with 100 μL and once with 200 μL of ice-cold binding buffer, the cell-bound radioactivity was measured using a gamma counter (Packard Cobra II, GMI, Ramsey, MN, USA). The 50% inhibitory concentration (IC_50_) values were calculated by fitting the data using a nonlinear regression algorithm (GraphPad Software). Experiments were performed three times.

Internalization experiments were performed as previously described [35]. Briefly, 10^5^ LNCaP or PC-3 cells were seeded in poly-l-lysine-coated 24-well cell culture plates 24 h before incubation. After washing with PBS, the cells were incubated with the radiolabeled compounds ^68^Ga]6 or ^68^Ga]7 (25 nM final concentration) for 45 min at 37°C and at 4°C, respectively. To determine specific cellular uptake, cells were blocked with 2-(phosphonomethyl)-pentanedioic acid (PMPA, Axxora, Loerrach, Germany) to a final concentration of 100 μM. Cellular uptake was terminated by washing four times with 1 mL of ice-cold PBS. To remove surface-bound radioactivity, cells were incubated twice with 0.5 mL glycine-HCl in PBS (50 mM, pH = 2.8) for 5 min. The cells were washed with 1 mL of ice-cold PBS and lysed using 0.3 N NaOH (0.5 mL). The surface-bound and the internalized fractions were measured in a gamma counter.

### Cell surface retention

The determination of cell surface retention was performed in accordance to a previously described experiment [36]. Briefly, 5 × 10^6^ LNCaP cells in a 320-μL RPMI 1640 medium were incubated with ^68^Ga]6 or ^68^Ga]7 (25 nM final concentration), respectively, for 45 min at 37°C. Cells were washed three times and resuspended in the RPMI 1640 medium (320 μL); samples were taken as a control for total cell-associated activity (cell surface bound and internalized). Subsequently, 0.5 μL of a 2-PMPA solution (100 mmol/L in DMSO) was added to avoid rebinding and to enhance the competitive pressure on releasing the radioactivity from the cell surface. At indicated time points, 10-μL samples were taken and transferred on top of a 400-μL microcentrifuge tube containing 350 μL of a 75:25 mixture of silicon oil (density 1.05; Aldrich), and mineral oil (density 0.872; Acros, Thermo Fisher Scientific) [37]. Subsequently, the tubes were centrifuged at 12,000 rpm for 2 min to separate cellular radioactivity and radioactivity solved in the medium. After freezing in liquid nitrogen, the bottom tips containing the cell pellet were cut off. The cell pellets and the medium were measured in a gamma counter to determine cellular uptake

(1) $$\left(\%\;\text{uptake}=\frac{100\times \text{cpminpellet}}{\text{cpminpellet}+\text{cpminsupernatant}}\right)$$

### Biodistribution and PET imaging

Male BALB/c nu/nu mice of 7 to 8 weeks old (Charles River Laboratories, Wilmington, MA, USA) were subcutaneously inoculated into the right trunk with 5 × 10^6^ cells of either LNCaP or PC-3 (both in 50% Matrigel; Becton Dickinson, Heidelberg, Germany). The tumors were allowed to grow for 3 to 4 weeks until approximately 1 cm^3^ in size. The ^68^Ga-radiolabeled compounds were injected into a tail vein (1 to 2 MBq/mouse; 0.1 to 0.2 nmol). At 1 h after injection, the animals were sacrificed. Organs of interest were dissected, blotted dry, and weighed. The radioactivity was measured using a gamma counter and calculated as percentage injected dose per gram (% ID/g).

For the microPET studies, 10 to 25 MBq of either [^68^Ga]6 or [^68^Ga]7 in a volume of 0.15 mL (approximately 0.5 nmol) was injected via a lateral tail vein into mice bearing LNCaP tumor xenografts. The anesthetized animals (2% sevoflurane, Abbott, Wiesbaden, Germany) were placed in prone position into an Inveon small animal PET scanner (Siemens, Knoxville, TN, USA) to perform a 50-min dynamic microPET scan starting at 1 min post-injection followed by a 20-min static scan. All animal experiments complied with the current laws of the Federal Republic of Germany.

### Statistical aspects

All experiments were performed at least in triplicate. Quantitative data were expressed as mean ± standard deviation (SD). If applicable, means were compared using Student's *t* test. *P* values <0.05 were considered statistically significant.

## Results

### Synthesis of the monomeric and dimeric PSMA inhibitors

The synthesis of intermediate 5, which exposes an unprotected amino group for further coupling of the HBED-CC active esters, was carried out by solid-phase chemistry (Figure 1). The monomeric reference 6, previously described and characterized [32], was formed by reacting the monoactive ester of HBED-CC with the protected binding motif 5. The dimer 7 was obtained from a reaction of bis-activated HBED-CC-TFP ester with 5. Analytical data of the HBED-CC conjugates 6 and 7 are summarized in Table 1.

### Radiolabeling and stability

Low precursor amounts and low concentrations of compounds 6 or 7 (0.1 nmol; 1.7 μM) were sufficient to gain RCYs of >99% after a 2-min reaction time at room temperature. Specific activities of >500 GBq/μmol could be obtained for both formats. As a consequence of high RCY, the biological testing was performed without further purification of the radioligands. The analytical data are summarized in Table 1.

The stability of the ^68^Ga-labeled compounds 6 and 7 was investigated in human serum for 2 h at 37°C and in PBS in the presence of 10 μM transferrin. Both assays proved the stability of the compounds with respect to hydrolysis and *trans*-complexation. After 2 h of incubation, HPLC analysis revealed 99% intact [^68^ Ga]6 and [^68^Ga]7. Furthermore, no changes in size exclusion chromatography were detected after incubation in human serum or in the presence of *apo*-transferrin.

### Determination of PSMA affinity

In order to determine the binding affinity, the variants were competitively analyzed in an enzyme-based assay on rhPSMA (NAALADase assay) and in a cell binding assay with LNCaP cells using ^67^Ga-labeled 6 (radiochemical purity > 99%, specific activity approximately 1,000 GBq/μmol) as radioligand. The affinity-related IC_50_ values of both assays indicated an improved affinity of at least a factor of 3 of the dimeric compound [^nat^Ga]7 as compared to the monomer [^nat^Ga]6 (*P* < 0.05). The IC_50_ values are summarized in Table 1.

### Internalization and efflux

**Figure 2:**
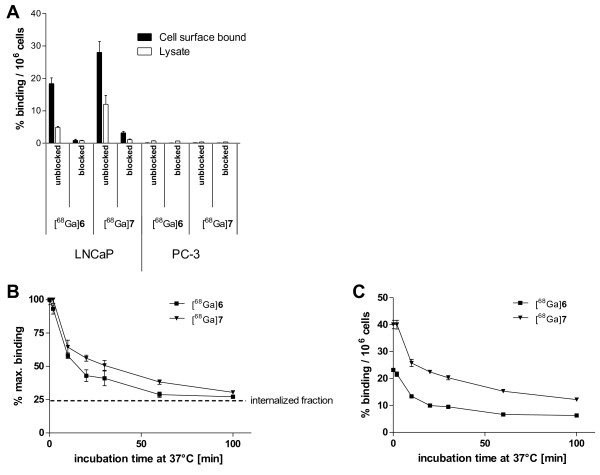
**Cell binding and internalization of [**^**68**^**Ga]6 and [**^**68**^**Ga]7 (A)**. Specific cellular uptake was evaluated by blockage using 100 μM 2-PMPA. (**B**) The graph shows the release of radioactivity from cells in percentage of initially bound compound. (**C**) The release of radioactivity related to the initial cellular uptake derived from A. Values in A and C are expressed as percentage of applied radioactivity bound to 10^6^ cells. Data are expressed as mean ± SD (*n* = 3).

Internalization experiments on LNCaP and PC-3 cells indicated specific cell surface binding and internalization of [^68^Ga]6 and [^68^Ga]7 (Figure 2a). The uptake in PSMA^+^ LNCaP cells was blocked in the presence of 2-PMPA, and both compounds showed no binding to PSMA^−^ PC-3 cells at all. The dimeric compound [^68^Ga]7 significantly showed higher cellular uptake (40.04 ± 6.09% (dimer), 23.24 ± 2.17% (monomer); *P* = 0.01), while both substances were internalized to approximately 25%. This results in a higher absolute amount of internalized radioactivity using the dimer. The efflux experiment showed prolonged cell surface retention of the dimeric compound (Figure 2b), while the lower plateau of both dissociation curves seems to represent the internalized fraction of the compounds, indicating that only surface-bound radioactivity is released from the cells. As shown in Figure 2b, the monomer reached the internalized level earlier, resulting in higher overall cellular uptake of the dimer at all time points (Figure 2c).

### Biodistribution and PET imaging

**Figure 3:**
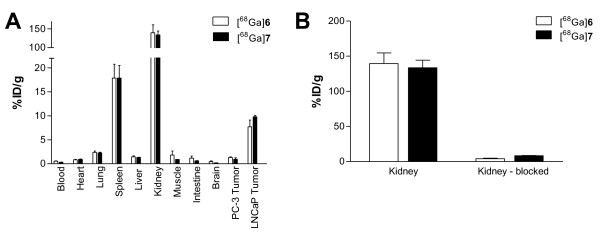
**Organ distribution expressed as % ID/g tissue 1 h post-injection.** (**A**) Comparison of the monomer [^68^Ga]**6** and the dimer [^68^Ga]**7**. (**B**) PSMA blocking by co-administration of 2 mg of 2-PMPA/kg body weight. Data are expressed as mean ± SD (*n* = 5).

The biodistribution data are summarized in Table 2 and Figure 3a. Both, the monomeric compound [^68^Ga]6 and the dimeric compound [^68^Ga]7, were cleared rapidly from the circulation and PSMA^−^ tissues. In comparison to the monomer [^68^Ga]6, the dimeric compound [^68^Ga]7 showed no higher uptake in PSMA^−^ organs like the lung or intestine at 1 h post-injection. The liver uptake of both compounds was low (1.43 ± 0.19% ID/g ([^68^Ga]6), 1.29 ± 0.11% ID/g ([^68^Ga]7)).

**Table 2:** **Organ distribution (1 h post-injection) of [**^**68**^**Ga]6 and [**^**68**^**Ga]7 in BALB/c nu/nu mice**

**Organ**	**Monomer [**^**68**^**Ga]6**	**Dimer [**^**68**^**Ga]7**
Blood	0.53 ± 0.04	0.31 ± 0.03
Heart	0.83 ± 0.08	0.90 ± 0.12
Lung	2.36 ± 0.27	2.25 ± 0.24
Spleen	17.90 ± 2.87	17.88 ± 4.53
Liver	1.43 ± 0.19	1.29 ± 0.11
Kidney	139.44 ± 21.40	133.43 ± 18.88
Muscle	1.00 ± 0.24	0.90 ± 0.01
Intestine	1.14 ± 0.46	0.57 ± 0.14
Brain	0.40 ± 0.19	0.17 ± 0.01
PC-3 tumor	1.30 ± 0.12	0.93 ± 0.53
LNCaP tumor (*n* = 5)	4.89 ± 1.34	8.22 ± 1.78

Injection dose was 1 to 2 MBq/mouse, 0.1 to 0.2 nmol. Data are expressed as % ID/g ± SD, *n* = 3.

The kidney uptake of 139.44 ± 21.40% ID/g ([^68^Ga]6) and 133.43 ± 18.88% ID/g ([^68^Ga]7) could be blocked after the co-injection of 2 mg/kg of PMPA to 4.02 ± 1.14% ID/g ([^68^Ga]6) and 8.29 ± 0.83% ID/g ([^68^Ga]7), respectively (Figure 3b). The spleen uptake was similarly reduced after blocking, 17.90 ± 2.87% ID/g ([^68^Ga]6) and 17.88 ± 4.53% ID/g ([^68^Ga]7) vs. 1.54 ± 0.33% ID/g ([^68^Ga]6) and 0.67 ± 0.11% ID/g ([^68^Ga]7), respectively. The tumor uptake was 8.22 ± 1.78% ID/g for the dimer and 4.89 ± 1.34% ID/g for the monomer 1 h p.i. (*P* = 0.02). Remarkably, the dimer shows a significant lower uptake in the blood (*P* = 0.002) and a trend to lower background activity (Table 2). Together with the faster blood clearance, the improved tumor uptake of the dimer resulted in a considerable higher tumor-to-blood ratio of 26.5 (T/B_monomer_ = 9.2).

**Figure 4:**
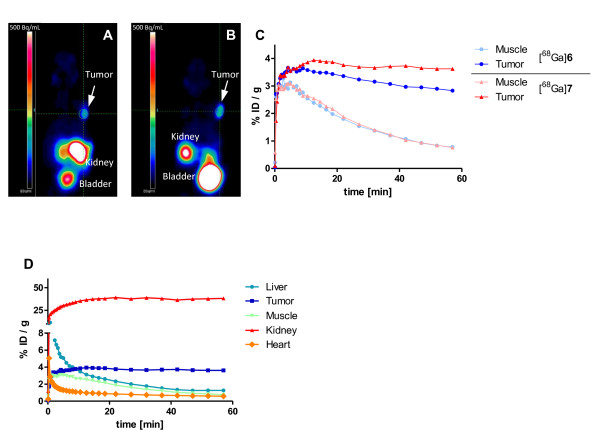
**Whole-body coronal microPET image of an athymic male nude mice bearing LNCaP tumor xenograft.** The monomer [^68^Ga]**6** (**A**) and the dimer [^68^Ga]**7** (**B**) were evaluated by a dynamic microPET scan followed by a static scan. The static scans 1 h post-injection of [^68^Ga]**6** and [^68^Ga]**7** are shown in (A) and (B), respectively. Approximately 15 MBq/mouse was injected. (**C**) The graph shows the respective time-activity curves in the muscle and tumor for both tracers. (**D**) The graph demonstrates the elimination of [^68^Ga]**7** from other organs in PET.

The microPET coronal slices of tumor-bearing mice shown in Figure 4a,b indicate the enhanced tumor uptake of [^68^Ga]7. The microPET time-activity curves (Figure 4c) clearly demonstrate improved tumor retention of the dimer while observing comparable muscle washout of both compounds. The time-activity curves in Figure 4d show rapid elimination of radioactivity from important organs, proving the early formation of high tumor-to-background contrast.

**Figure 5:**
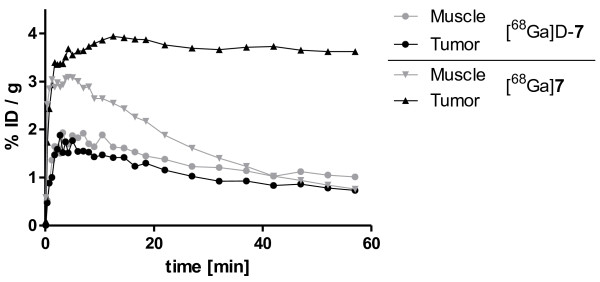
**Time-activity curves of [**^**68**^**Ga]7 and [**^**68**^**Ga]**d**-7 taken from dynamic PET measurements and expressed as % ID/g.**

The PSMA specificity of both tracers was confirmed by investigating the organ distribution with PSMA^−^ PC-3 tumor-bearing mice. The tumor uptake values amounted to 1.30 ± 0.12% ID/g ([^68^Ga]6) and 0.93 ± 0.53% ID/g ([^68^Ga]7) (Figure 3a). Additional demonstration of the *in vivo* specificity of the dimeric PSMA inhibitor [^68^Ga]7 was obtained by the comparison with the respective d-isomer [^68^Ga]d-7 (Figure 5). [^68^Ga]d-7 shows considerably reduced binding capacity to PSMA-expressing LNCaP cells as compared to the l-form [^68^Ga]7 confirmed by competitive cell binding experiments (*K*_i_(d-7) > 5 μM). As a consequence, the l-isomer showed accumulation in the tumor, whereas the d-isomer is cleared as rapidly as from the muscle.

## Discussion

PET imaging with ^18^ F]FDG, radiolabeled choline, and acetate is hampered by the generally low glycolytic rate and slow growth of most prostate tumors [38,39]. Tracers binding to overexpressed cell surface receptors such as PSMA are more favorable since they bind prostate cancer cells independent of proliferation [15-17]. Furthermore, PSMA exhibits the potential to be effectively internalized via clathrin-coated pits and subsequent endocytosis [40] which leads to enhanced tumor cell retention of the radiotracer and, accordingly, to a better image quality.

Here, we describe the dimerization of Glu‐ureido‐Lys, the PSMA-targeting pharmacophore, using the heterobifunctional chelator HBED-CC. The pharmacophore represents three carboxylic groups interacting with the respective side chains of PSMA, one oxygen being part of the zinc complex in the active center, and as provided by HBED-CC, an aromatic structure element which is able to interact with the hydrophobic part of the enzyme composed of tyrosines [31,32]. Molecules lacking one of these interactions showed other modes of action resulting in weaker binding or dramatically reduced internalization rates [31]. As a consequence, both the determination of affinity and the internalization rate are crucial for the characterization of PSMA inhibitors. Considering the particular characteristics of the PSMA binding pocket [31], especially, the internalization rate might be influenced by two hydrophilic pharmacophores on both sides of the HBED-CC scaffold. Since the affinity was significantly enhanced and the internalization rate has not been negatively affected, the dimer shows an improved interaction with the active binding site. As the chelator is simultaneously used as dimerization scaffold, complexing moiety, and lipophilic part, ^68^Ga]7 represents a radiometal-labeled dimeric PSMA inhibitor with exceptionally low molecular size. This is considered as an important issue since the size of a radiopharmaceutical tracer is often crucial in terms of pharmacokinetic properties.

Dimerization is a suitable methodology to enhance the binding properties of tumor-targeting tracers. For example, cyclic RGD dimers, such as E[c(RGDfK)]_2_, were developed as diagnostic and therapeutic radiotracers with improved binding and imaging properties [27,41]. However, the improvement of binding capacity by dimerization is often accompanied by higher kidney or liver uptake of the tracer [27]. Thus, dimerization can unfavorably influence pharmacokinetics or targeting properties, resulting in less advantageous tumor-to-background ratios [42,43]. Our results did not indicate unfavorable influences on pharmacokinetics as a consequence of dimerization. The PSMA-specific HBED-CC dimer ^68^Ga]7 is cleared even faster than the respective monomer ^68^Ga]6 and showed nearly no unspecific liver uptake. A blocking experiment showed that the initial high kidney uptake was mainly PSMA specific. The improved background clearance of the dimer and the enhanced tumor uptake (a factor of approximately 1.7) resulted in a considerably improved tumor-to-blood ratio (T/B_dimer_ = 26.5; T/B_monomer_ = 9.2) at 1 h post-injection.

In order to characterize *in vitro* properties, the affinity, cellular uptake, and cell surface retention of the molecules were studied. The affinity constants determined on both, the purified receptor and LNCaP cells, indicated significantly higher affinity of the dimer. Since higher affinity of dimeric compounds is often caused by reduced dissociation rates, the cell surface retention of both compounds has been studied in a cell surface retention experiment. To avoid rebinding of released radioactivity, 2-PMPA was added as a competitor during the experiment. As a consequence, it was possible to detect the internalized fraction level (Figure 2b) beyond which the gallium complexes are no longer released. The released radioactivity is, therefore, only related to the cell surface-bound activity.

The prolonged cell surface retention of the dimer is in agreement with the higher affinity of [^68^Ga]7, indicating that the dissociation rate was improved by dimerization. The higher amount of cell surface-bound radioactivity and the prolonged contact with the cell surface resulted in a higher absolute amount of internalized radioactivity.

The excellent PSMA specificity of the probes found in cell binding assays was demonstrated *in vivo* by comparing the organ distribution data using PSMA^+^ (LNCaP) and PSMA^−^ (PC-3) tumor xenografts. Additional proof of specificity was obtained with a dimer containing d-Glu. The time-activity curves obtained from dynamic PET measurements of this nearly non-binding isomer [^68^Ga]d-7 demonstrated the large difference in tumor uptake (Figure 5).

Another proof of specificity of the PSMA-binding ^68^Ga complexes was obtained through the blockade of the high kidney and spleen uptake values with co-injected 2-PMPA (Figure 3b), indicating that the uptake was mainly mediated by specific binding to physiologically expressed PSMA in those murine organs [44-47].

A limitation of this study seems to be the high kidney uptake of the investigated monomer and dimer. There are numerous examples of variably labeled urea-based molecules with extremely high murine kidney uptake [18,19,23,48,49]. It was shown in these and in our studies that the uptake is mainly specific as a consequence of physiologically expressed PSMA in murine kidneys [18,32]. With regard to clinical translation, however, it should be mentioned that PSMA expression is lower in human kidneys [50], leading to encouraging clinical results with those tracers [33].

## Conclusions

Taken together, it could be demonstrated that the [^68^Ga]7 complex shows an improved PSMA affinity, higher cell uptake, and prolonged cell surface retention as compared to the monomeric [^68^Ga]6. The enhanced tumor uptake and retention together with the faster washout from background organs resulted in a higher tumor-to-background ratio. These promising features favor the application of the dimer for PET imaging with the short-lived ^68^Ga. The convenient labeling technology might influence the introduction into clinical routine, helping to improve the diagnosis of recurrent prostate cancer.

## Competing interests

The authors declare that they have no competing interests.

## Authors’ contributions

MS participated in the conception and design of the study and carried out the peptide/chemical synthesis. UBW carried out the *in vitro* experiments and contributed in the analysis and interpretation of data. FZ was involved in the chemical characterization of the molecules and analysis. KL carried out the *in vivo* experiments and analysis. WM, UH, and MiE contributed in revising the manuscript critically. MaE contributed in the conception and design of the study, in the analysis and interpretation of data, and in the final approval of the manuscript. The manuscript has been seen and approved by all involved authors.
